# The Role of Physical Activity for the Management of Sarcopenia in People Living with HIV

**DOI:** 10.3390/ijerph17041283

**Published:** 2020-02-17

**Authors:** Matteo Bonato, Filippo Turrini, Laura Galli, Giuseppe Banfi, Paola Cinque

**Affiliations:** 1 IRCSS Istituto Ortopedico Galeazzi, Via Riccardo Galeazzi 4, 20161 Milan, Italy; banfi.giuseppe@hsr.it; 2 IRCCS San Raffaele Scientific Institute, Via Olgettina 60, 20132 Milan, Italy; turrini.filippo@unisr.it (F.T.); galli.laura@hsr.it (L.G.); cinque.paola@hsr.it (P.C.); 3 Vita-Salute San Raffaele University, Via Olgettina 58, 20132 Milan, Italy

**Keywords:** muscle, HIV, cART, immune activation, orthopaedic

## Abstract

Sarcopenia is a physiopathological process associated with aging, caused by reduction of muscle strength, muscle quality and physical performance, and associated with an increased risk of falls, physical disability and premature death. There is no effective treatment for sarcopenia, but physical exercise seems to be highly effective at counteracting the decline in muscle mass and strength associated with aging. Recently, sarcopenia has been recognized as an emerging issue in people living with HIV (PLWH). Despite adequate treatment with combination antiretroviral therapy (cART), PLWH may exhibit an early occurrence of some aging-related conditions, including sarcopenia, frailty and falls, and this is likely resulting from high rates of comorbidities, high-risk behaviours, chronic immune activation and cART-specific factors. In this review, we discuss the potential mechanisms and the clinical relevance of sarcopenia in PLWH, and present data from longitudinal studies of physical activity in this population. Despite none of these studies having specifically addressed the benefits of physical exercise on sarcopenia, there is evidence that exercise is effective to increase aerobic capacity and muscle strength, and to improve body composition and inflammatory outcomes in PLWH. Therefore, the expected benefits of physical exercise are likely to translate into a successful and specific intervention for prevention and treatment of sarcopenia in this population.

## 1. Introduction

Sarcopenia is defined by the progressive reduction of muscle mass, muscle strength and function occurring in the elderly and in people with chronic conditions, such as metabolic syndrome, cardiovascular disease or cancer [1]. Starting from the age of thirty, our body faces a slow natural loss of muscle tissue. However, this process is accelerated in the elderly and in people with some pathological conditions. Its prevalence in the elderly population is largely variable, ranging from 5% to 50% depending on age, gender, pathological conditions and diagnostic criteria [2]. As examples, a large study of community-dwelling older people from United Kingdom—mean age 67 years—showed a prevalence of sarcopenia of 4.6% in men and 7.9% in women [3], whereas an earlier large survey in New Mexico documented an increased overall prevalence with age, from below 24% in women and men below 70 years of age to >50% in those over 80 [4].

Sarcopenia is clinically relevant because it is associated with an increased frequency of adverse health outcomes, such as frailty, functional disabilities, falls and, in general, quality-of-life impairment [5,6,7]. There is no effective treatment for sarcopenia, but physical exercise seems to be highly effective at counteracting the decline in muscle mass and strength associated with aging. Based on the evidence that resistance exercise increases muscle mass and strength, a recent position paper from the Society of Sarcopenia Cachexia and Wasting disorders (SCWD) recommended resistance exercise to any older persons for both secondary prevention and/or treatment of sarcopenia [8].

Sarcopenia is an emerging health issue in people living with HIV (PLWH). After more than three decades since the beginning of the AIDS epidemic, and following the introduction of combination antiretroviral therapy (cART), HIV infection has now become a chronic and manageable condition [9]. Consequently, the average life span of PLWH approaches today that of the general population [10]. However, the combination of aging with chronic inflammation, lifestyle factors and cART toxicities has contributed to an increasing risk of developing chronic diseases, also including sarcopenia, frailty and falls [11]. There are only few data on the prevalence of sarcopenia in PLWH, although, similarly to other chronic diseases, this syndrome seems to occur more frequently and at an earlier age than in the general population.

The aim of this review is to discuss the mechanisms and the clinical relevance of sarcopenia in PLWH, to describe the efficacy of physical activity interventions on muscle mass and function, and to provide evidence towards an effective treatment approach for sarcopenia in this population.

## 2. Mechanisms of Sarcopenia in People Living with HIV

A number of factors play a role in the development of sarcopenia in the general population, including lack of physical exercise, hormonal changes, nutritional deficiencies and low protein intake, metabolic alterations and chronic inflammation [12]. Among these risk factors, the presence of metabolic alterations and increased immune activation are key features of HIV infection in cART-treated persons. Chronic immune activation is characterized by an increased release of proinflammatory mediators, presence of dysfunctional T-regulatory cells and a T-cell-senescent phenotype. These alterations are also observed in the elderly, overall defining the conditions of “immunosenescence” and “inflammaging” [13]. Immune activation in PLWH may result from the persistence of HIV infection in latently infected cells, but other factors are likely to contribute, including cART, risk behaviours, e.g., smoking or use of drugs, and other comorbidities [14]. In turn, chronic inflammation is one of the most important risk factors for the developing of non-AIDS diseases, including cardiovascular, kidney or liver disease, cancers, some neurological diseases and “geriatric syndromes”, including sarcopenia, frailty and falls [15].

**Figure 1:**
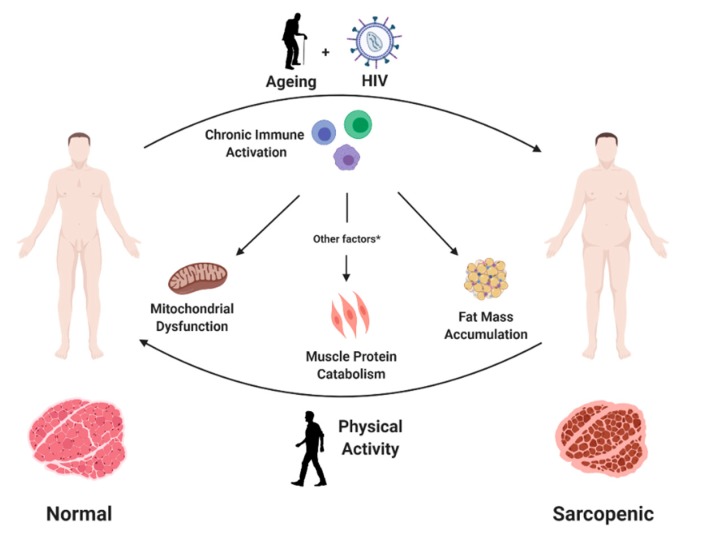
Persistent HIV infection and ageing contribute to development of sarcopenia in people with HIV. Chronic immune activation, common to both conditions, may favour this process through mitochondrial dysfunction, increased muscle protein catabolism and muscle fat accumulation. Other factors include a low protein intake and vitamin D deficiency, among others. In contrast, physical exercise may prevent and partly reduce sarcopenia through increasing muscle mass and function and reducing chronic inflammation.

Sarcopenia is characterized by a progressive loss of muscle fibres that are replaced by adipose tissue, increasing fibrosis and changes in muscle metabolism [16]. Several mechanisms have been suggested to explain how persistent inflammation may lead to these changes in the muscular tissue (Figure 1). A first potential mechanism involves mitochondrial dysfunction. Immune activation is known to increase reactive oxygen species (ROS) intracellular concentration and cause redox balance disturbances [17], which, in turn, may lead to mitochondrial DNA damage due to its proximity to free radical sources and the relative lack of a protein scaffold. Consequently, mitochondrial DNA mutations can impair mitochondrial protein synthesis, determining loss of oxidative phosphorylation efficiency and, ultimately, premature cell senescence [18].

Another consequence of increased immune activation is the accumulation of macrophages and other immune cells in the adipose tissue, with an increased release of pro-inflammatory cytokines and adipokines [19]. These inflammatory mediators may favour lipid accumulation and trigger protein catabolism with loss of muscle mass and further increase local inflammation [20].

Some traditional risk factors may also specifically promote sarcopenia progression in PLWH, including a low vitamin D level [21] and factors associated with lifestyle [22]. The relevance of vitamin D for skeletal muscle metabolism has been highlighted in recent years, and ageing is associated with vitamin D deficiency likely resulting from decreased sun exposure and ability to synthesize Vitamin D, and reduced expression of vitamin D receptor in muscle tissue [21]. Vitamin D deficiency seems indeed to be more prevalent in PLWH compared to the general population, possibly associated with immune activation, exposure to specific antiretroviral drugs and high prevalence of metabolic diseases [23]; low-function subjects had a greater frequency of vitamin D deficiency compared to high-function subjects [24]. Among factors associated with lifestyle, cigarette smoking is also more prevalent in PLWH than in the general population [25] and it may also increase the chance of developing sarcopenia [22].

## 3. Epidemiological and Clinical Aspects of Sarcopenia in People Living with HIV

According to the original 2010 consensus of the European Working Group on Sarcopenia in Older People (EWGSOP) on the definition and diagnosis of sarcopenia [26] and the updated 2019 version [1], the operational definition of sarcopenia is based on the assessment of three specific aspects: (i) muscle strength, as measured by handgrip strength or a chair-stand test; (ii) muscle quality, as measured by total body or appendicular skeletal muscle mass by magnetic resonance imaging (MRI), computer tomography (CT) or dual-energy X-ray absorptiometry (DEXA), and further adjustable for height, weight or body mass index; and (iii) physical performance, as determined by measuring gait speed, e.g., during a six-minute walking test, or by using other composite tests, including walking, balance and chair-standing exercises. Sarcopenia is diagnosed in the presence of low values for the first two criteria, whereas it is considered severe when also the third criterion is met [1]. In PLWH, a large number of studies have assessed the prevalence and described the characteristics of one or more of these parameters taken separately, which were usually altered compared to the general population [9,27]. For instance, low muscle strength and impairment of physical performance are often found to be impaired in PLWH and these parameters are indeed used in the definition of frailty, another common syndrome in people ageing with HIV [27,28,29]. On the other hand, the assessment of muscle quality and volume by CT showed significantly lower muscle density and greater fat infiltration in PLWH compared to HIV-uninfected controls [30], whereas DEXA studies of body composition show that treated HIV-infected persons gain more fat, but lose lean mass over time compared with HIV-uninfected persons [31].

However, only few studies have reported the prevalence of sarcopenia as a syndrome resulting from the combination of the above measures in treated PLWH [27,32,33]. The Multicenter AIDS Cohort Study (MACS) showed a prevalence of 17% among 185 men with HIV, median age 60, with no differences compared to HIV-negative people of the same age [27]. Conversely, prevalence was 24% in a smaller Brazilian study [32], enrolling 33 HIV-positive persons, mean age 59 years old, with a 4.95 higher risk compared to older—mean age 70 years old—HIV-negative controls. Similarly, the prevalence of sarcopenia among 153 HIV-infected Asians was higher in those above 50 years of age compared to HIV-uninfected controls matched by age, sex and ethnicity (17% vs. 4%), but not significantly different when people below the age of 50 were included (10 vs. 6%) [33]. In a large Spanish cohort of PLWH assessed in the period 2000–2016, the overall prevalence of sarcopenia in those aged >50 years was 27.8%, with a different gender distribution, involving 43% of women and 8.8% of men [34].

Some of these studies also identified specific risk factors for sarcopenia in PLWH, in addition to traditional risk factors such as female gender, increasing age and high body mass index. These included low education level and employment status, long duration of HIV infection, low CD4^+^ T-cell counts at the start of cART and long-time exposure to certain nucleoside reverse transcriptase inhibitors (NRTIs), i.e., zidovudine, stavudine or didanosine [27,33,34]. Finally, specific health outcomes were also analysed in relation to the presence of sarcopenia, including mortality risk scores, quality of life, healthcare utilization, functional disability and falls. Out of these, sarcopenia was associated with a five-fold higher mortality scores and a four-fold higher risk of functional disability [27].

## 4. Exercise Intervention to Counteract Sarcopenia in PLWH

Sedentary lifestyle is one of the principal causes for loss of muscle mass and strength, which, in turn, determines further reduction of activity levels with further muscle weakness [35]. In contrast, regular physical exercise is highly effective at counteracting the decline in muscle mass and strength and, possibly, also in reducing the chronic inflammation associated with aging.

Indeed, physical activity represents the most effective strategy in the management of sarcopenia in the general population and in specific patient groups [36]. However, no study has specifically addressed the benefits of physical activity on sarcopenia in PLWH. There are several differences in sarcopenia between PLWH and the general elderly population, and the benefits of physical activity may also differ. PLWH may show a greater degree of immune activation and develop sarcopenia at an earlier age. In addition, the rate of sedentary lifestyle seems to be higher in PLWH than in the general population, as well as the frequency of risk behaviours such as smoking or drug use, which may limit adherence to exercise [37]. We have here reviewed the longitudinal interventional studies of physical activity in PLWH focusing on the outcomes that could be relevant for management of sarcopenia, i.e., physical fitness, body composition and inflammatory indexes (Table 1).

Physical activity interventions are usually followed by improvement in physical function, because of an increase in cardiorespiratory and muscular fitness [38]. Cardiorespiratory fitness (CRF) reflects the integrated ability of the human organism to transport oxygen from the atmosphere to the mitochondria to perform physical work. CRF depends on a linked chain of processes, including pulmonary ventilation and diffusion, ventricular function, ventricular–arterial coupling, ability of the vasculature to accommodate and efficiently transport blood from the heart to match oxygen requirements and ability of the muscle cells to receive and use the oxygen and nutrients delivered by the blood [38]. CRF thus quantifies the functional capacity of an individual and is considered a reflection of total body health. Physical activity contributes to improved CRF, approximately for 45%–50% [38]. The maximal oxygen uptake ($\dot{V}$O_2max_) is the gold standard for measuring the integrated cardiopulmonary-muscle oxidative function, and studies using the $\dot{V}$O_2max_ as fitness outcome have shown that and adequate physical exercise intervention invariably improves CRF in PLWH (Table 1).

Muscular fitness is a general term used to describe muscular performance in relation to strength, endurance and overall health. Muscular strength is measured in (i) dynamic strength: measure of the maximum weight that can be lifted once (1 Repetition Maximum, 1RM); (ii) static strength: measure of the maximum force that one can apply to an unmoving object (e.g., handgrip strength); and (iii) muscular endurance: measured through multiple lifting repetitions using weights that are below one’s maximum capacity. Muscular strength and endurance improve when muscle fibres grow stronger and new muscles form, and when the supply of oxygen and energy to the muscles becomes more efficient. In PLWH, several studies have proven that adequate resistance training exercises is successful in improving muscular strength, as assessed by using all of the above approaches (Table 1).

In longitudinal studies of physical activity in PLWH, the combination of improved cardiovascular and muscular fitness was associated with other health outcomes with relevance on sarcopenia, including improvements in body composition and inflammatory markers. In particular, a number of studies reported a reduction in fat mass and an increase in fat-free mass, using either dual-energy X ray absorptiometry, computed tomography or bio-impedentiometry. These results were observed irrespectively of type of exercise (endurance or resistance, or a combination of both), its duration and frequency (from 20 to 60 min for 2/3 times a week) and the duration of the study (from 6 to 48 weeks). A few recent studies have also investigated the effects of physical activity on inflammatory outcomes in PLWH. Most of these showed a reduction of soluble markers of inflammation, such as high-sensitivity C-reactive protein, Interleukin-6 and Interleukin-8 (Table 1). Additionally, physical exercise—both endurance and combined exercise training—was also followed by a marked decrease of the frequency of CD8+/CD38+/HLA-DR+ activated T-cells [60].

## 5. Conclusions

PLWH may show a degree of musculoskeletal impairment and an increased risk of sarcopenia, likely resulting from a combination of HIV-associated chronic immune activation, metabolic complications and advancing ageing. Different physical activity interventions have shown to be effective to increase muscle mass and function in this population. However, no study has so far addressed sarcopenia as specific target of physical exercise intervention in PLWH. Nevertheless, physical activity, in its various blends, increases aerobic capacity and muscle strength and this is associated with improvement in body composition and inflammatory outcomes. The information from longitudinal trials of physical exercise is relevant in order to prescribe cost-effective tailored exercise interventions and protocols that focus specifically for prevention and treatment of sarcopenia in PLWH.

## Figures and Tables

**Table 1:** Longitudinal interventional studies that assessed the effect of physical activity on physical fitness, body composition and inflammatory markers in cART-treated people living with HIV.

Author	Participants	Age	Intervention	Outcomes		
				Physical Fitness	Body Composition	Inflammatory Markers
Roubenoff et al. [39]	10 subjects with lipodystrophy	39.2 (23–56) Mean (range)	Duration 16 weeks Frequency: 3 sessions/week Exercise combined - *Endurance*: 20 min bike - *Resistance*: 1 h at 80% 1 RM	↑Leg press; ↑Leg extension; ↑Chest press.	↓Total Body fat; ↓Trunk fat	n.a.
Agin et al. [40]	37 women allocated in: 12 protein supplementations 12 progressive resistance training 12 combined treatment	40.8 (29–55) Mean (range)	Duration: 14 weeks Frequency: 3 sessions/week Exercise: - *Resistance*: 3 × 8/10 at 75% 1RM	*Protein supplementation* ↑Bench press; ↑Shoulder press; ↑Biceps curl; ↑Triceps extension; ↑Leg extension. *Resistance training* ↑Bench press; ↑Seated back row; ↑Shoulder press; ↑Biceps curl; ↑Triceps extension; ↑Leg extension; ↑Leg curl *Combined treatment* ↑Bench press; ↑Seated row; ↑Shoulder press; ↑Biceps curl; ↑Leg extension; ↑Leg curl.	*Protein supplementation* ↑Body weight; ↑Fat mass; ↑Fat free mass *Resistance training* ↓Body cell mass; ↑Skeletal muscle; ↓Fat mass; ↓Fat free mass. *Combined treatment* ↓Body cell mass; ↑Fat free mass	n.a.
Yarasheski et al. [41]	18 subjects with hypertriglyceridemia	42 ± 2 Mean ± SD	Duration: 16 weeks Frequency: 4 sessions/week Exercise: - *Resistance*: 3 × 5/8 at 75/85% 1 RM	↑in muscle strength.	↑Body weight; ↑Lean mass; ↓Fat mass; ↓Thigh muscle area	n.a.
Roubenoff et al. [42]	10 HIV-seropositive wasted 12 HIV-seropositive nonwasted	38.9 (30–53) Mean (range)	Duration: 8 weeks Frequency: 3 sessions/week Exercise: - *Resistance*: 3 × 8 from 50% to 80% 1RM	*HIV wasting syndrome* ↑Chest press; ↑Leg press; ↑Leg extension; ↑Upper back. *No HIV wasting syndrome* ↑Chest press; ↑Leg press; ↑Leg extension; ↑Upper back.	*HIV wasting syndrome* ↑Fat free mass *No HIV wasting syndrome* ↑Fat free mass	n.a.
Thöni et al. [43]	17 subjects with lipodystrophy and 2 with dyslipidaemia	44.2 ± 2.3 Mean ± SD	Duration: 16 weeks Frequency: 2 sessions/week Exercise: *Endurance*: cycling 45 min at VT	↑$\dot{V}$O2max	↓Abdominal adipose tissue; ↓Visceral adipose tissue	n.a.
Driscoll et al. [44,45]	37 subjects with hyperinsulinemia and fat redistribution allocated in: 19 metformin and exercise 18 metformin only	42 (35–27) Median (IQR)	Duration: 12 weeks Frequency: 2 sessions/week Exercise: combined - *Endurance*: 20/30 min cycling at 60%–75% HRmax - *Resistance*: 3 × 10 at 60%–80% 1RM	*Metformin and exercise* ↑Exercise time	*Metformin and exercise* ↓Waist-to-hip ratio; ↓Abdominal subcutaneous fat; ↑Thigh muscle area.	n.a.
Engelson et al. [46]	39 obese women	41.8 ± 7.5 Mean ± SD	Duration: 12 weeks Frequency: 3 sessions/week Exercise: combined - *Endurance*: 30 min running at 70%–80% HRmax - *Resistance*: 3 × 8/10 at 70% 1RM	↑Pectoral; ↑Latissimus dorsi; ↑Quadriceps; ↑Time to fatigue.	↓Body mass; ↓BMI; ↓Waist circumference; ↓Chest circumference; ↓Biceps skinfold; ↓Abdominal skinfold; ↓Thigh skinfold; ↑Skeletal muscle; ↓Visceral adipose tissue; ↓Subcutaneous adipose tissue; ↓Total adipose tissue; ↓Fat mass	n.a.
Terry et al. [47]	42 subjects with dyslipidaemia and lipodystrophy allocated in: 21 diet and exercise group 21 diet group	36 ± 6 Mean ± SD	Duration: 12 weeks Frequency: 3 sessions/week Exercise: - *Endurance*: 30 min running at 70%–85% HRmax	*Exercise and diet* ↑$\dot{V}$O2max	*In both groups* ↓Body mass; ↓BMI; ↓Waist-to-hip ratio; ↓Body fat	n.a.
Dolan et al. [48]	40 women with fat redistribution allocated in: 20 exercise group 20 control group	42 ± 2 Mean ± SEM	Duration: 16 weeks Frequency: 3 sessions/week Exercise: combined - *Endurance*: 20/30 min cycling at 60%–75% HRmax - *Resistance*: 3/4 × 8/10 at 60%–80% 1RM	*Exercise* ↑$\dot{V}$O2max; ↑Time to exercise; ↑Strength; ↑6MWT; ↑Knee extensor; ↑Knee flexor; ↑Pectoralis; Shoulder abductors; ↑Ankle plantar flexor; ↑Elbow flexors	*Exercise* ↓Waist circumference; ↓Total muscle area	n.a.
Robinson et al. [49]	9 subjects with HIV metabolic abnormalities	44.0 ± 3.8 Mean ± SD	Duration: 16 weeks Frequency: 3 sessions/week Exercise: combined - *Endurance*: 20 min bike at 70%–80% $\dot{V}$O2max - *Resistance*: 1 × 10 at 60%–80% 1RM	↑1RM in all strength exercises	↓Trunk fat	n.a.
Hand et al. [50]	74 subjects allocated in: 44 exercise group 30 control group	41.8 ± 3.7 Mean ± SD	Duration: 6 weeks Frequency: 3 sessions/week Exercise: combined - *Endurance*: 30 min at 50%–70% $\dot{V}$O2max - *Resistance*: 20 min at 60% 3RM	*Exercise training* ↑Time to fatigue; ↑Estimated $\dot{V}$O2max; ↓Functional aerobic impairment; ↑HRpeak during exercise; ↓Submaximal exercise	n.a.	n.a.
Lindegaard et al. [51]	20 men with lipodystrophy allocated in: 10 endurance group 10 strength group	49.5 ± 8.2 Mean ± SD	Duration:16 weeks Frequency: 3 sessions/week Exercise: - Endurance: 35 min at 65%–75% $\dot{V}$O2max - Resistance: 3 × 8 at 50%–80% 3RM	*Endurance training* ↑$\dot{V}$O2max; ↑3RM *Strength training* ↑3RM	*Strength training* ↓Body mass; ↑Total lean mass; ↓Total fat mass; ↓Trunk fat mass; ↓Limb fat mass	*Endurance training* ↓hsCRP; ↓TNF-α; ↓IL6; ↓IL18 *Strength training* ↓IL18; ↔hsCRP; ↔TNF-α; ↔IL6.
Farinatti et al. [52]	27 subjects allocated in: 19 experimental group 8 control group	44 ± 4 Mean ± SD	Duration: 12 weeks Frequency: 3 sessions/week Exercise: combined - *Endurance*: 30 min at 150 bpm - *Resistance*: 3 × 12 at 60–80 12RM - Flexibility: 3 × 30 s	*Exercise training* ↑Flexibility; ↑Leg press; ↑Seated row; ↓HR during exercise	*Exercise training* ↔ Body Mass; BMI	n.a.
Souza et al. [53]	26 women subjects allocated in: 11 living with HIV 21 controls	65.65 ± 3.06 Mean ± SD	Duration: 48 weeks Frequency: 2 sessions/week Exercise: - *Resistance*: 3 × 8/12	*Exercise with HIV* ↓Sit standing; ↑2.4 miles walking; ↑Leg press; ↑Chest press; ↑Lumbar extension; ↑Seated row; ↑Seated abdominal.	*Exercise with HIV* ↔ Body Mass; BMI	n.a.
Dudgeon et al. [54]	111 men allocated in: 59 exercise group 59 control group	44.9 ± 1.4 Mean ± SD	Duration: 6 weeks Frequency: 2 sessions/week Exercise: combined - *Endurance*: 30 min at 60%–75% HRmax - *Resistance*: 3 × 12 circuit training	n.a.	*Exercise* ↑Total lean mass ↔ Body mass; ↔ BMI; ↔ Total body fat; %Trunk fat; ↔ Arm fat; ↔ Leg fat; ↔ Arm/leg/trunk lean mass	*Exercise* ↓Salivary cortisol; ↔ IL6; ↔ IL1; ↔ sTNFrII; ↔ IGF1; ↔ IGFBP3; ↔ GH; ↔ Salivary testosterone
Broholom et al. [55]	20 subjects with lipodystrophy allocated in: 10 endurance group 10 strength group	49.5 ± 10.3 Mean ± SD	Duration: 16 weeks Frequency: 3 sessions/week Exercise: - *Endurance*: 35 min interval training - *Resistance*: 3 × 8/10 rep at 50–80 3 RM	*Endurance* ↑$\dot{V}$O2max; ↑3RM *Strength* ↑3RM	n.a.	
Ezema et al. [56]	33 subjects allocated in: 17 exercise 16 control	36.27 ± 10.06 Mean ± SD	Duration: 8 weeks Frequency: 3 sessions/week Exercise: - *Endurance*: 45–60 min at 60%–79% HRmax	*Exercise* ↑$\dot{V}$O2max	*Exercise* ↔ BMI	
Ahmad et al. [57]	8 subjects	38 ± 9 Mean ± SD	Individual marathon running plan	↑Maximal running velocity; ↑VT	↔ BMI	
Garcia et al. [58]	10 subjects	44.7 ± 8.9 Mean ± SD	Duration: 20 weeks Frequency: 3 sessions/week Duration: combined - *Endurance*: 30 min at 60%–75% $\dot{V}$O2max - *Resistance*: circuit training	n.a.	↑Lean mass; ↔ Body mass; ↔ BMI; ↔ Fat mass;	
Zanetti et al. [59]	30 subjects allocated in 15 non-linear resistance training 15 control	42.4 ± 10.5 Mean ± SD	Duration: 12 weeks Frequency: 3 times/week Exercise: - *Resistance*: 3/4 sets from 4 to 20 reps	*Non-linear resistance training* ↑Squat; ↑Bench press; ↑Hamstring curls; ↑Frontal pull; ↑Seated calf raise; ↑Shoulder press	*Non-linear resistance training* ↓Central subcutaneous fat; ↓Peripheral subcutaneous fat; ↓Total subcutaneous fat; ↓Neck circumference; ↓Abdomen circumference; ↓Waist circumference; ↓Waist-to-hip ratio	*Non-linear resistance training* ↓IL-1β; ↓IL6; ↓IL8; ↓IL10; ↓TNF-α
Bonato et al. [60]	49 subjects allocated in: 29 walk 20 strength-walk	48 (44–54) Median (IQR)	Duration: 12 weeks Frequency: 3 sessions/week Duration: combined - *Endurance*: 60 min at 65%–75% HRmax - *Resistance*: 3 × 12 circuit training	*Walk* ↑6MWT *Strength-walk* ↑6MWT; ↑1RM strength in all circuit training exercises	*Walk* ↓Body mass; ↓Body mass index	*Walk* ↓CRP; ↓IL6; ↓D-dimer *Strength-walk* ↓CRP
Pedro et al. [61]	28 subjects allocated in: 11 training group 17 control group	45.1 ± 7.7 Mean ± SD	Duration: 16 weeks Frequency: 3 sessions/week Exercise: combined - *Endurance*: 20 min at 50%–70% HRR - *Resistance*: 2/3 sets × 8/12 rep	*Concurrent training* ↑Leg press; ↑Bench press	n.a.	*Concurrent training* ↓IL8
Zanetti et al. [62]	21 subjects with metabolic syndrome allocated in: 10 non-linear resistance training 11 control	41.1 ± 10.1 Mean ± SD	Duration: 12 weeks Frequency: 3 times/week Exercise: - *Resistance*: 3/4 sets from 4 to 20 reps	n.a.	*Non-linear resistance training* ↓Fat mass; ↑Lean mass; ↓Waist circumference;	n.a.
Oursler et al. [63]	22 subjects allocated in: 11 moderate-intensity 11 high-intensity	57.4 ± 9.5 Mean ± SD	Duration: 16 weeks Frequency: 3 sessions/week Exercise: endurance - *Moderate-intensity training*: 45 min self-paced walking - *high-intensity training*: 45 min at 75%–90% HRR.	*Moderate-intensity training* ↑Time on treadmill; ↑6MWT *High-intensity training* ↑$\dot{V}$O2peak; ↑Time on treadmill; ↑6MWT	*Moderate-intensity training* ↔ Body mass; ↔ Lean Mass; ↔ Fat mass; ↔ Body fat percent; ↔ Visceral fat area; ↔ Subcutaneous fat area *High-intensity training* ↔ Body mass; ↔ Lean Mass; ↔ Fat mass; ↔ Body fat percent; ↔ Visceral fat area; ↔ Subcutaneous fat area	n.a.
Vingren et al. [64]	16 men with substance abuse treatment 8 resistance training 8 control	42 ± 11 Mean ± SD	Duration: 6 weeks Frequency: 3 sessions/week Exercise: - *Resistance*: 3/5 × 5/12 reps	*Resistance training group* ↑1RM strength at bench press; ↑Iso squat peak; ↑Vertical power	*Resistance training group* ↑Muscle mass; ↑Upper-arm circumference; ↑Forearm circumference	*Resistance training group* ↔ IFNγ, ↔ IL1β, ↔ IL2, ↔ IL4, ↔ IL6, ↔ IL10, ↔ TNF-α
De Brito-Neto et al. [65]	19 subjects allocated in 9 exercise group 10 control group	39.16 ± 5.11 Mean ± SD	Duration: 12 weeks Frequency: 2 sessions/week Exercise: - *Resistance*: 3 × 18/10 reps at subjective perception of effort	*Exercise group* ↓Bench press; ↓Lat pulldown; ↓Triceps pulley; ↓Elbow flexion; ↓Squat; ↓Leg press; ↓Knee flexion; ↓Ankle extension.	*Exercise group* ↓Body fat; ↑Lean body mass	n.a.
Zanetti et al. [66]	82 subjects allocated in: 21 placebo 21 statins 21 placebo + exercise training 20 statins + exercise training	41.9 ± 10.4 Mean ± SD	Duration: 12 weeks Frequency: 3 sessions/week Exercise: - *Endurance*: periodized polarized training - *Resistance*: periodized nonlinear resistance training	*Placebo + exercise training* ↑$\dot{V}$O2max; ↑Bench press; ↑Hamstring curl; ↑Frontal pull; ↑Seated calf raise; ↑Shoulder press *Statins + exercise training* ↑$\dot{V}$O2max; ↑Bench press; ↑Hamstring curl; ↑Frontal pull; ↑Seated calf raise; ↑Shoulder press	*Placebo + exercise training* ↑Lean body mass; ↓Fat mass *Statins + exercise training* ↑Lean body mass; ↓Fat mass	*Statins* ↓IL-1β; ↓IL6; ↓hsCRP; ↓FIBR; *Placebo + exercise training* ↓IL-1β; ↓IL6; ↓IL10; ↓CRP; ↓FIBR; *Statins + exercise training* ↓IL1β; ↓IL6; ↓IL8; ↓IL10; ↓CRP; ↓FIBR;
Bonato et al. [67]	38 subjects allocated in: 20 APP 18 No-APP	51 (44–54) Median (IQR)	Duration: 16 weeks Frequency: 3 sessions/week Exercise: - *Endurance*: 60 min at 65%–75% HRmax	*APP* ↑$\dot{V}$O2peak	*APP* ↓Fat mass; ↑Fat free mass	n.a.

*Notes:* Reps: repetitions; RM: repetition maximum; SD: standard deviation; SEM: standard error of the mean; IQR: 25%–75% interquartile range; VT: ventilatory threshold; $\dot{V}O_{2\max}$: maximal oxygen uptake; $\dot{V}O_{2\mathrm{peak}}$: peak oxygen uptake; HR: heart rate; HRR: heart rate reserve; bpm: beats per minutes; HIV: human immunodeficiency virus; 6MWT: six-minute walking test; BMI: body mass index; IL: interleukin; hsCRP: high sensitivity c reactive protein; FIBR: fibrinogen; TNF-α: tumor necrosis factor alpha; IGF1; insulin-like growth factor 1; IGFBP3; insulin-like growth factor-binding protein 3; APP: training program provided by a smartphone application; No-APP: training provided by a hard-copy training program; n.a.: not applicable.
